# A CRM-Integrated ypT Staging System Improves Prognostic Stratification Following Neoadjuvant Therapy in Rectal Cancer

**DOI:** 10.7150/jca.129779

**Published:** 2026-03-17

**Authors:** Wan-Hsuan Chow, Chung-Han Ho, Yi-Chen Chen, Hsuan-Yi Huang, Ching-Chieh Yang

**Affiliations:** 1 Department of Radiation Oncology, Chi Mei Medical Center, Tainan, Taiwan.; 2 Department of Medical Research, Chi Mei Medical Center, Tainan, Taiwan.; 3 Department of Information Management, Southern Taiwan University of Science and Technology, Tainan, Taiwan.; 4 Division of Colorectal Surgery, Department of Surgery, Chi Mei Medical Center, Tainan, Taiwan.; 5 Center of General Education, Chia Nan University of Pharmacy and Science, Tainan, Taiwan.; 6 Department of Pharmacy, Chia-Nan University of Pharmacy and Science, Tainan, Taiwan.; 7 School of Medicine, College of Medicine, National Sun Yat-sen University, Kaohsiung, Taiwan.

**Keywords:** rectal cancer, neoadjuvant chemoradiotherapy, CRM, ypT, survival.

## Abstract

**Introduction:**

The ypT staging system has limited prognostic value after neoadjuvant therapy, as it primarily reflects only tumor characteristics alone. This study proposes a novel staging system that integrates circumferential resection margin (CRM) status with the ypT category to enhance prognostic accuracy following neoadjuvant chemoradiotherapy (nCRT) for rectal cancer.

**Methods:**

We analyzed data from 4,308 rectal adenocarcinoma patients treated with nCRT followed by surgery, using the Taiwan Cancer Registry and National Health Insurance Research Database (2011-2021). CRM involvement was defined as a margin ≤1 mm. Overall survival was assessed using multivariable Cox regression, and prognostic performance of the proposed CRM-integrated ypT staging system was compared with the American Joint Committee on Cancer (AJCC) TNM system using Harrell's c-statistic.

**Results:**

CRM involvement (≤1 mm) was significantly associated with worse 5-year survival (adjusted odds ratio, 0.44; 95% CI, 0.31-0.61). Due to the low rate of CRM positivity in ypT0-2 patients, a modified ypT classification was established: new ypT3 (ypT3 and CRM-), new ypT4A (ypT4A and CRM-), new ypT4B (ypT3 and CRM+ or ypT4B and CRM-), and new ypT4C (ypT4A and CRM+ or ypT4B and CRM+). This system demonstrated better prognostic discrimination than the current AJCC classification (Harrell's c-statistic: 0.756 vs. 0.752, P = 0.034).

**Conclusions:**

Incorporating CRM into the ypT stage offers survival stratification and may guide more individualized postoperative treatment strategies for rectal cancer patients after nCRT.

## 1. Introduction

Surgical resection is a cornerstone of curative therapy for rectal cancer. Introduced in the early 1980s, total mesorectal excision (TME) has become the surgical standard, leading to marked improvements in local control and long-term survival 1-3. However, surgery alone is often insufficient for optimal tumor control, especially in cases involving bulky tumors or lesions that extend toward the mesorectal fascia, where achieving a clear circumferential resection margin (CRM) can be technically challenging. This limitation has driven the widespread adoption of neoadjuvant chemoradiotherapy (nCRT), which enhances local control and reduces perioperative morbidity 4-8. Consequently, this treatment paradigm has not only improved surgical outcomes but also shifted clinical emphasis toward pathological features, including post-treatment tumor and nodal status (ypT/ypN), tumor regression grade (TRG), and CRM.

Among these, CRM status is one of the most extensively validated predictors of both local recurrence and long-term survival 9-12. Liu et al., analyzing a large national cohort of rectal cancer patients undergoing surgery, demonstrated that a CRM ≤ 1 mm was independently associated with nearly double the risk of cancer-specific mortality compared with margins of 1.1-2.0 mm (HR = 1.99, 95% CI = 1.61-2.45, P < 0.001), underscoring the importance of achieving a clear CRM at resection 9. Similarly, Park et al. reported that among various CRM thresholds, ≤ 1 mm provided the strongest prognostic discrimination for disease-free survival in both chemoradiotherapy and non-chemoradiotherapy groups 10. In a meta-analysis of 75 studies including over 85,000 patients published between 2006 and 2019, Detering et al. found that CRM involvement was significantly associated with inferior 5-year outcomes: local recurrence (OR = 4.67), disease-free survival (OR = 3.63), distant metastasis (OR = 2.95), and overall survival (OR = 3.21), even in the era of modern surgical and multidisciplinary treatment 11. More recently, in a propensity-matched cohort of stage II/III rectal cancer patients, CRM involvement was again associated with markedly poorer outcomes, with 5-year recurrence-free survival of 15.7% versus 57.9% (HR = 2.72, P = 0.004) and cancer-specific survival of 35.3% versus 71.3% (HR = 3.52, P = 0.002) 12.

The prognosis and treatment strategies for rectal cancer are largely guided by the American Joint Committee on Cancer (AJCC) TNM classification. However, the current system lacks sufficient precision in outcome prediction, as it primarily reflects tumor characteristics alone 13. Patients within the same stage often experience markedly different outcomes, underscoring the need to refine the TNM staging system to better identify individuals at high risk of recurrence or death. Previous studies have established CRM as both a marker of surgical quality and an independent prognostic factor. Therefore, integrating CRM with the ypT classification may provide clinicians with more accurate prognostic information. This study investigates whether incorporating CRM into the ypT category can improve survival risk stratification in rectal cancer patients treated with nCRT.

## 2. Materials and Methods

### 2.1 Data source and study cohort

**Figure 1:**
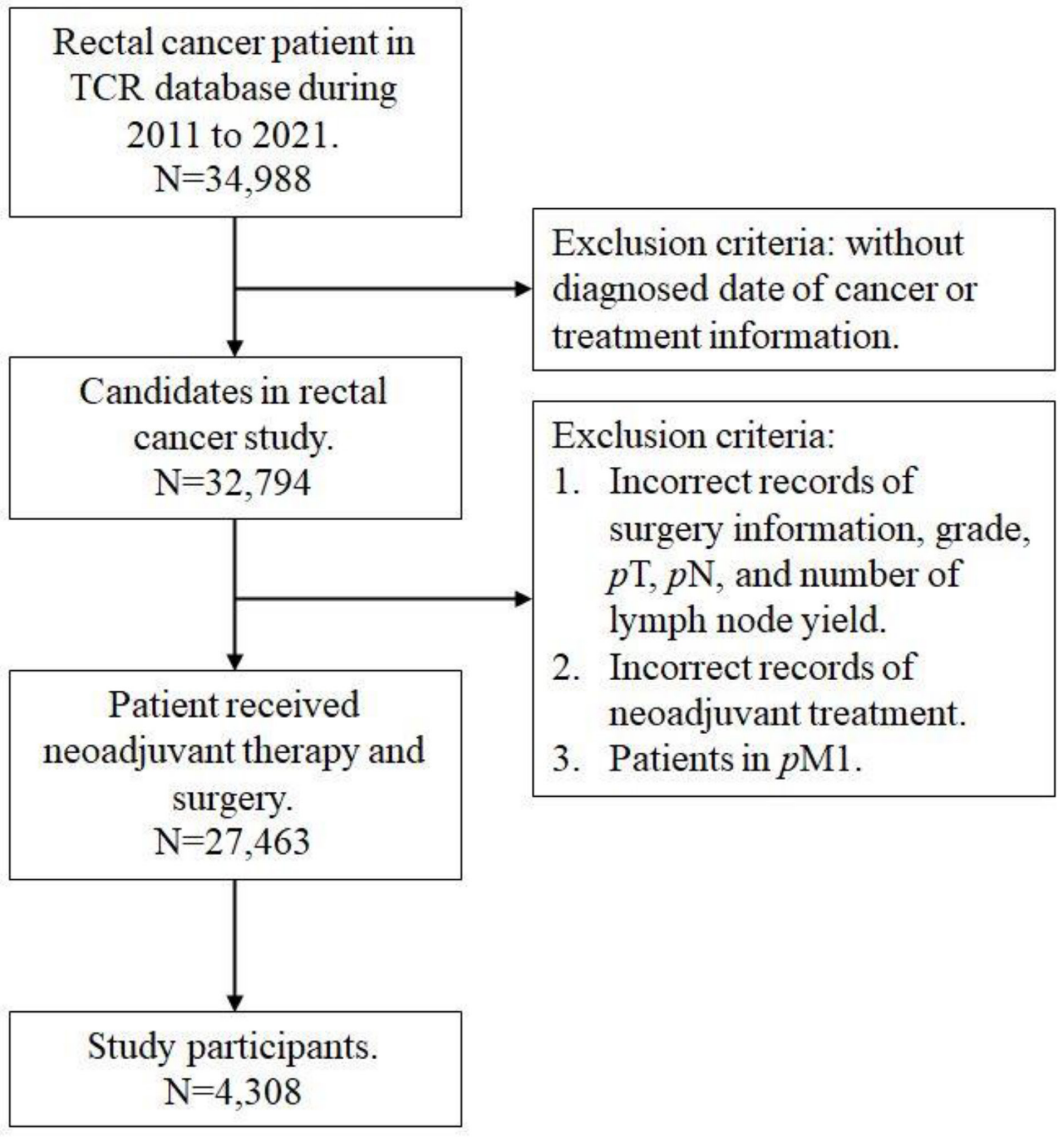
Flow chart of study participant.

This study was designed to assess the effect of CRM on survival of rectal cancer patients. We retrieved data from the national TCR and NHIRD between January 2011 and December 2021, which captures almost all cancer patients in Taiwan. Because the NHI institute employed an accuracy program to verify the quality of coding, the TCR database has been approved as excellent and complete 14. According to the International Classification of Disease for Oncology, third edition codes, the study cohort included patients diagnosed with topography codes for 'rectum' (C20.9) and histology codes for 'adenocarcinoma' (8140, 8210, 8263, and 8280) 15. The staging for all cases was defined based from the 8th edition American Joint Committee on Cancer (AJCC) classification system 16. Collected demographic and clinicopathological variables included age, sex, clinical and pathological tumor and nodal category, grade, surgery type, CRM, lymph node yield, lymph node ratio, comorbid status, and cancer-related treatment. Surgical procedures were categorized as low anterior resection, abdominoperineal resection, and others which included pull-through with sphincter preservation, proctectomy or proctocolectomy with en bloc resection of adjacent organs, or local tumor excision/total proctocolectomy. The CRM, recorded to the nearest tenth of a millimeter in the pathology report, represents the distance between the tumor's leading edge and the nearest surgically dissected margin, as defined by the AJCC Seventh Edition Cancer Staging Manual as the non-peritonealized surface of the specimen. A CRM ≤ 1 mm is defined as positive in our database and selected for analysis. The Charlson Comorbidity Index (CCI) score was employed to quantify the complexity of comorbid conditions 17. Patients were excluded if they had (1) a prior history of cancer, including non-rectal malignancies, or metastatic disease identified intraoperatively and pathologically confirmed, or (2) incomplete or unclear coding of clinicopathological or treatment information. Finally, a total of 4308 rectal cancer patients received nCRT and surgery were included in our study participants (Figure 1).

### 2.2 Statistical Analysis

#### 2.2.1Patient information

Demographic and clinicopathological in-formation were presented with descriptive statistic. The primary endpoint was 5-year overall survival, calculated from the date of cancer diagnosis rather than the date of surgery. All statistical analyses were performed using SAS 9.4 for Windows (SAS Institute, Inc., Cary, NC, USA), and Stata version 15 (Stata Corp, College Station, Texas, USA). Statistical significance was set at P < 0.05.

#### 2.2.2 Identification and handling of CRM-inclusive ypT staging system

By using a logistic regression model, the estimated effect between survival rate, ypT, CRM, and those prognostic factors, was presented as odd ratios (ORs) with 95% confidence intervals (95% CIs). Prognostic factors potentially related to the survival that were significant on univariable analysis were entered into the multivariable analysis. According to the study aim, we modified ypT category incorporating CRM status to assess the association between survival and achieving a clear margin in CRM-positive patients. Survival rate stratified by modified ypT category incorporating CRM status displayed a monotonically decreasing trend, and the Cochran-Armitage trend test was used to test the ordinal trend in the newly defined staging system 18. To compare the prognostic discrimination of the CRM-inclusive ypT staging system versus the traditional ypT classification, we applied Harrell's concordance index (C-index) to compare the performance of the respective staging categories 19. In addition, cumulative survival rates in the newly defined staging system were also used to evaluate the time-dependent survival predictive performance, by using the Kaplan-Meier method, and the differences between curves were derived from the Log-rank test. Heagerty's integrated area under the curve (iAUC) analysis estimates the time-dependent predictive performance using a rank-based method for the followed-up survival 20. The C-index and iAUC, ranging from 0.5 (no discrimination) to 1.0 (perfect discrimination), is used to assess predictive accuracy in survival prediction.

#### 2.2.3 CRM-inclusive ypT staging system validation

Internal validation was performed using bootstrapping to assess potential optimism in the performance of the CRM-inclusive ypT staging system. A total of 500 bootstrap samples were generated from the original dataset, with stepwise variable selection repeated in each sample. This nonparametric approach estimates prediction error and provides stable, assuming the study population represents a random sample of rectal cancer patients 21. External validation was conducted using an independent dataset to evaluate the consistency of staging system performance, including patient in different ypN and lymph node yield.

## 3. Results

### 3.1 Patient Characteristics

The baseline characteristics of the study cohort are summarized in Table 1. A total of 4,308 rectal cancer patients treated with neoadjuvant chemoradiotherapy followed by surgery were included, comprising 3,046 males (70.7%) and 1,262 females (29.3%), with a mean age of 60.4 ± 11.7 years and a mean follow-up of 3.5 ± 1.6 years. Clinical tumor status (cT) was cT1 in 13 (0.3%), cT2 in 508 (11.8%), cT3 in 3,169 (73.9%), and cT4 in 601 (14.0%) patients. Clinical nodal status (cN) was cN0 in 1,037 (24.1%), cN1 in 1,583 (36.8%), and cN2 in 1,677 (39.0%). After nCRT and surgery, pathological tumor status (ypT) was ypT0 in 756 (17.6%), ypT1 in 204 (4.7%), ypT2 in 1,037 (24.1%), ypT3 in 2,118 (49.2%), and ypT4 in 193 (4.5%). Pathological nodal status (ypN) was ypN0 in 2,908 (67.5%), ypN1 in 1,035 (24.0%), and ypN2 in 365 (8.5%). Most patients (74.9%) underwent low anterior resection, 64.8% had a lymph node yield ≥12, and 2,948 (68.4%) received adjuvant chemotherapy. CRM positivity (≤1 mm) was observed in 208 patients (4.8%). Survival outcomes by baseline characteristics are shown in Supplementary Table 1.

### 3.2 Modified ypT staging system in combination with CRM

In both univariable and multivariable analyses, CRM positivity was significantly associated with lower 5-year survival and remained an independent prognostic factor after adjustment for clinicopathologic variables (adjusted odds ratio [aOR] 0.44; 95% CI: 0.31-0.61; Table 2). Based on these findings, we developed a modified staging system combining ypT category and CRM (Table 3). The new prognostic staging system was defined as follows: new ypT3 (ypT3 and CRM-), new ypT4A (ypT4A and CRM-), new ypT4B (ypT3 and CRM+ or ypT4B and CRM-), and new ypT4C (ypT4A and CRM+ or ypT4B and CRM+). Patients with ypT0-2 were not reclassified in the modified system due to the small number with CRM positivity; only four patients (ypT0: n = 1; ypT1: n = 1; ypT2: n = 2) had CRM-positive status. The new system showed a stepwise decline in 5-year survival from 73.5% (new ypT3) to 59.6% (new ypT4A), 46.8% (new ypT4B), and 41.7% (new ypT4C), compared with 71.8% (ypT3) and 48.5% (ypT4) in the AJCC system. Overall, the new system demonstrated superior prognostic discrimination, with a higher C-index (0.756 vs. 0.752, P = 0.034; Table 4) and better curve separation (Supplementary Figure 1).

**Figure 2:**
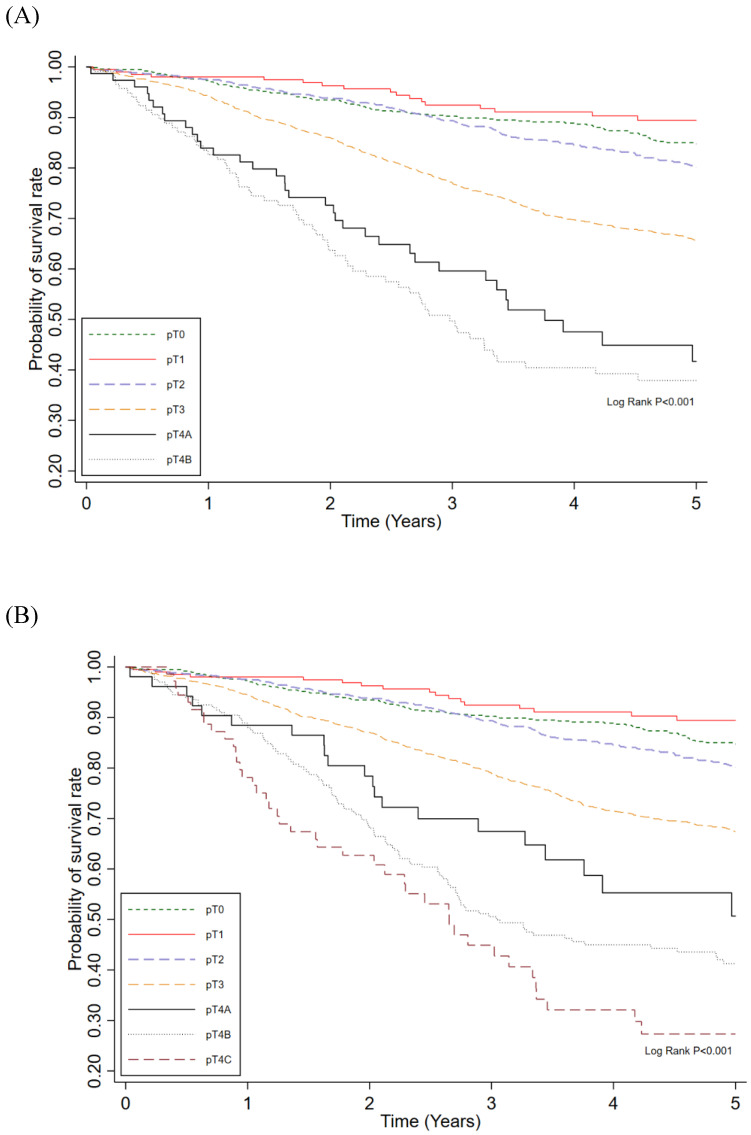
Probability of survival rate among ypT category **(A)** and new category pT **(B)** in rectal cancer patients treated with neoadjuvant chemoradiotherapy and surgery.

**Figure 3:**
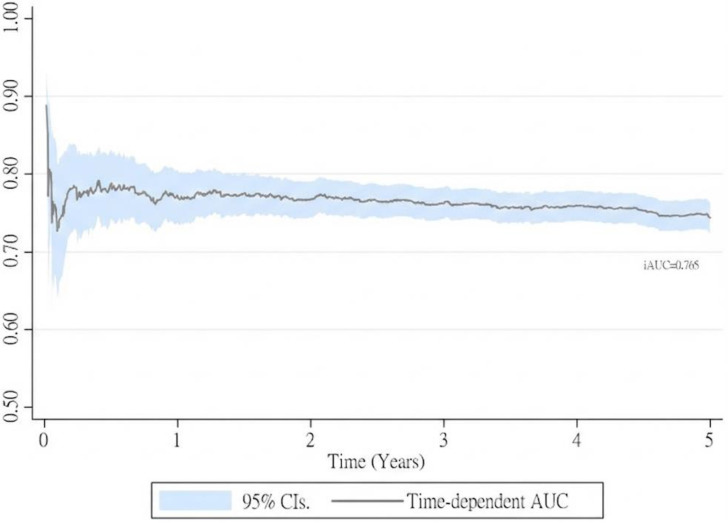
The performance of new category pT derived from adjusted cox regression in rectal cancer patients treated with neoadjuvant chemoradiotherapy and surgery.

To assess the time-dependent predictive performance of the new prognostic staging system, Kaplan-Meier survival curves were plotted for 5-year survival according to the AJCC ypT staging system (Figure 2A) and the new system (Figure 2B). The new staging system demonstrated more distinct separation of survival curves, with differences between stages reaching statistical significance (P < 0.01). In addition, the cumulative predictive performance of the new system yielded an iAUC of 0.765 over the 5-year follow-up period (Figure 3).

### 3.3CRM-inclusive ypT staging system validation

The performance of the CRM-inclusive ypT staging system during 500-sample bootstrap validation is presented in Table 5. Internal bootstrap validation yielded a C-index of 0.756 (95% CI, 0.720-0.791). We further evaluated the performance of this revised ypT category across different nodal statuses (ypN and lymph node yield), and it consistently demonstrated excellent predictive ability. Taken together, our findings indicate that incorporating CRM into the conventional AJCC ypT staging system improves the accuracy of 5-year survival prognostication in rectal cancer patients treated with nCRT and surgery.

## 4. Discussion

We propose a novel ypT staging system for rectal cancer that incorporates CRM status to improve survival stratification. Using a large, population-based cohort from Taiwan's National Cancer Registry and National Health Insurance Research Database, we analyzed patients treated with nCRT followed by surgery. To our knowledge, this is the first study to formally integrate CRM into the ypT framework. The revised system produced more clearly separated survival curves, indicating improved prognostic discrimination, which was supported by a significantly higher Harrell's C-index compared with the conventional AJCC staging system (0.756 vs. 0.752; P = 0.034). The modest absolute improvement in the C-index likely reflects that patients with ypT0-2 disease were not reclassified in the modified system, as CRM positivity was uncommon in this subgroup. Accordingly, the added prognostic value of the new staging system was mainly observed among patients with more advanced ypT categories, in whom CRM status provided further risk stratification.

Although widely adopted after neoadjuvant therapy, the ypT staging system offers only modest prognostic value, as shown by Cui et al., who found that ypT stage was not independently associated with disease-free survival in multivariate analysis 13. A similar concern was raised by Min et al., who observed that patients with identical ypT stages could still experience differing survival outcomes depending on their tumor regression grade, particularly in the absence of nodal involvement 22. This highlights how ypT staging fails to reflect the degree of histologic tumor response following nCRT, which has been shown to correlate with long-term survival. Given the limitations of the current ypT category, increasing efforts have been made to incorporate additional prognostic factors into the staging system. Relevant prognostic variables include TRG, CRM, lymphovascular invasion (LVI), nodal status, and tumor budding 9-12, 22-26. KRAS mutations and radiologic evidence of EMVI have likewise been identified as prognostically relevant in recent studies 27-29. Several studies have investigated the integration of TRG into post-treatment staging systems to enhance predictive accuracy. For example, Cui et al. analyzed 329 patients treated with nCRT and surgery at a single institution, proposing the M-TTRG system—a composite metric that combines ypT and TRG into five prognostic groups 13. This model demonstrated superior stratification of 3-year disease-free survival compared to ypT or TRG alone. Similarly, Song et al. examined 331 patients with ypStage II/III disease and found that combining Dworak TRG (poor response: 1/2; good response: 3/4) with pathologic stage provided better prediction of 5-year disease-free survival, yielding a higher C-statistic than the conventional ypStage (0.784 vs. 0.757; P = 0.012) 30. However, TRG assessment is inherently subjective and prone to interobserver variability, which may limit its consistency in clinical application. In contrast to these TRG-based models, our study is the first to integrate CRM status directly into the ypT staging system. By integrating an anatomically defined and consistently reported margin parameter, our CRM-integrated system enhances reproducibility and clinical applicability, making it a more practical tool for routine risk assessment and treatment planning.

CRM involvement is a well-established prognostic factor in rectal cancer, strongly associated with both recurrence and overall survival 9-12. This association persists even in patients receiving nCRT. In a large cohort study, Park et al. reported that CRM ≤ 1 mm independently predicted worse outcomes in both nCRT and non-nCRT groups, with 5-year disease-free survival declining from 82.8% to 54.7% in non-nCRT patients and from 88.9% to 55.5% in those receiving nCRT (P < 0.001 and P = 0.001, respectively) 10. Despite its consistent prognostic relevance, CRM status has yet to substantially influence adjuvant treatment decisions and is still primarily considered a marker of surgical quality and an adverse factor guiding postoperative management.

A similar concept has been demonstrated in head and neck squamous cell carcinoma (HNSCC), where the AJCC 8th edition incorporated extranodal extension (ENE) into the nodal category. ENE has been consistently validated as a strong adverse prognostic factor across multiple cohorts, leading to proposals for revised nodal stratification. Moreover, RTOG 9501 and EORTC 22931 showed that ENE-positive patients benefit more from concurrent chemoradiotherapy than radiotherapy alone, highlighting how key risk features can shape staging and treatment decisions 31. Similarly, our results suggest that CRM involvement—a well-documented prognostic factor in rectal cancer—may warrant inclusion in future staging systems to enhance risk stratification and guide postoperative management.

This refined stratification enabled clearer identification of patients with unfavorable prognosis—those who might benefit from intensified adjuvant therapy or alternative systemic approaches. The conventional AJCC ypT classification, which separates ypT3 from ypT4 disease with estimated 5-year survival rates of 71.8% and 48.5%, respectively, offers only a broad risk distinction and may overlook meaningful differences among tumors with similar invasion depth. In contrast, our CRM-integrated model delineated four prognostically distinct subgroups, with 5-year survival ranging from 73.5% in new ypT3 to 41.7% in new ypT4C. This finer gradation in survival outcomes emphasizes the prognostic value of CRM and strengthens its potential role in guiding postoperative therapy selection and informing the use of novel treatment strategies, including consideration of more intensive adjuvant systemic therapy regimens or closer surveillance schedules for higher-risk subgroups.

This study has several limitations. First, CRM status was analyzed using a binary cutoff of ≤1 mm, and we did not explore alternative thresholds or continuous measurements, which may have further refined prognostic stratification. Second, in our cancer registry database, CRM assessment was limited to pathological findings instead of radiologist reading, and detailed information distinguishing positivity from tumor deposits versus direct tumor extension was not available. Future studies are warranted to address these limitations. Third, although we accounted for whether adjuvant therapy was administered, detailed information on chemotherapy regimens, dosing, treatment adherence, and molecular markers such as KRAS mutations was not available, which limited our ability to evaluate the impact of systemic treatment across subgroups. Finally, certain clinical details, such as surgical approach (open, laparoscopic, or robotic), were not available in the registry databases, and treatment strategies reflecting contemporary practice, including total neoadjuvant therapy (TNT), were not widely adopted during the study period, which may limit the generalizability of our findings to current clinical settings.

## 5. Conclusion

The findings from this study highlight the potential clinical utility of incorporating CRM status into ypT staging to improve risk stratification after neoadjuvant therapy. Recognizing its consistent association with survival outcomes, CRM holds potential for future incorporation of staging systems such as the AJCC, where its integration may offer a more clinically informative framework for guiding postoperative treatment decisions.

## Supplementary Material

Supplementary figure and table.

## Figures and Tables

**Table 1:** Baseline characteristic of rectal cancer patients treated with neoadjuvant chemoradiotherapy and surgery.

	N	%
Overall	4308	100.00
Age, years		
< 65	2761	64.09
≧ 65	1547	35.91
Sex		
Male	3046	70.71
Female	1262	29.29
Grade		
Well	2573	59.73
Moderately	152	3.53
Poorly	1583	36.75
cT category, missing n=17		
1	13	0.30
2	508	11.84
3	3169	73.85
4	601	14.01
cN category, missing n=11		
0	1037	24.13
1	1583	36.84
2	1677	39.03
ypT category		
0	756	17.55
1	204	4.74
2	1037	24.07
3	2118	49.16
4	193	4.48
ypN category		
0	2908	67.50
1	1035	24.03
2	365	8.47
Surgery type		
LAR	3228	74.93
APR	584	13.56
Others	496	11.51
CRM		
negative	4100	95.17
positive	208	4.83
LN removed number		
< 12	1518	35.24
≧ 12	2790	64.76
LNR group		
0-0.25	3926	91.13
0.26-0.50	241	5.59
0.56-0.75	91	2.11
0.76-1.00	50	1.16
Adjuvant treatment	2948	68.43
CCI score		
0	2756	63.97
1	866	20.10
≧ 2	686	15.92

Abbreviations: cT, clinical tumor; cN, clinical nodal; ypT, pathological tumor; ypN, pathological nodal; LAR, low anterior resection; APR, abdominoperineal resection; CRM, circumferential resection margin; LN, lymph node; LNR, lymph node ratio, CCI, Charlson Comorbidity Index.

**Table 2:** The risk factor of death in rectal cancer patients treated with neoadjuvant chemoradiotherapy and surgery.

Variable	Crude OR (95% CIs)	P-value	Adjusted OR (95% CIs)	P-value
ypT category				
0	Ref.		Ref.	
1	1.45 (0.85-2.49)	0.172	1.61 (0.92-2.80)	0.095
2	0.74 (0.56-0.97)	0.031	0.75 (0.56-1.00)	0.052
3	0.35 (0.27-0.44)	< .001	0.42 (0.33-0.55)	< .001
4				
T4A	0.15 (0.09-0.25)	< .001	0.22 (0.12-0.38)	< .001
T4B	0.12 (0.08-0.18)	< .001	0.16 (0.10-0.26)	< .001
CRM				
negative	Ref.		Ref.	
positive	0.22 (0.16-0.29)	< .001	0.44 (0.31-0.61)	< .001
Age, years				
< 65	Ref.		Ref.	
≧ 65	0.48 (0.41-0.55)	< .001	0.47 (0.40-0.55)	< .001
Sex				
Male	0.73 (0.62-0.86)	< .001	0.68 (0.57-0.82)	< .001
Female	Ref.		Ref.	
Grade				
Well	Ref.		Ref.	
Moderately	0.26 (0.19-0.37)	< .001	0.31 (0.22-0.46)	< .001
Poorly	0.98 (0.84-1.14)	0.802	1.01 (0.86-1.19)	0.909
ypN category				
0	Ref.		Ref.	
1	0.45 (0.38-0.53)	< .001	0.51 (0.42-0.62)	< .001
2	0.23 (0.19-0.29)	< .001	0.40 (0.27-0.61)	< .001
Surgery type				
LAR	Ref.		Ref.	
APR	0.49 (0.41-0.59)	< .001	0.59 (0.47-0.73)	< .001
Others	0.99 (0.79-1.25)	0.944	0.89 (0.69-1.15)	0.387
LNR group				
0-0.25	Ref.		Ref.	
0.26-0.50	0.34 (0.26-0.45)	< .001	0.83 (0.56-1.24)	0.363
0.56-0.75	0.28 (0.18-0.43)	< .001	0.75 (0.42-1.33)	0.329
0.76-1.00	0.14 (0.08-0.26)	< .001	0.39 (0.19-0.82)	0.013
Adjuvant treatment	1.43 (1.23-1.66)	< .001	1.98 (1.67-2.36)	< .001
CCI score				
0	Ref.		Ref.	
1	0.82 (0.68-0.98)	< .001	0.91 (0.75-1.12)	0.386
≧ 2	0.48 (0.40-0.58)	< .001	0.54 (0.44-0.66)	< .001

Abbreviations: ypT, pathological tumor; ypN, pathological nodal; LAR, low anterior resection; APR, abdominoperineal resection; CRM, circumferential resection margin; LNR, lymph node ratio, CCI, Charlson Comorbidity Index.

**Table 3:** The survival rates of rectal cancer patients according to different ypT category plus CRM combinations.

Category	ypT	CRM	Total patient	Survival, %	*P*-value
All patients					< .001
New T0	T0	-	756	666 (88.10)	
New T1	T1	-	204	187 (91.67)	
New T2	T2	-	1,037	876 (84.47)	
New T3	T3	CRM (-)	1,986	1,460 (73.51)	
New T4A	T4A	CRM (-)	52	31 (59.62)	
New T4B	T3	CRM (+)	201	94 (46.77)	
	T4B	CRM (-)			
New T4C	T4A	CRM (+)	72	30 (41.67)	
	T4B	CRM (+)			

*P*-value was derived from Cochran-Armitage Trend Test. Abbreviations: ypT, pathological tumor; CRM, circumferential resection margin.

**Table 4:** The performance between ypT and CRM-inclusive ypT staging system in rectal cancer patients treated with neoadjuvant chemoradiotherapy and surgery.

	*Model 1* ypT based				*Model 2* New category pT
Variable	Adjusted OR (95% CIs)	Variable			Adjusted OR (95% CIs)
ypT		New ypT	ypT	CRM	
0	Ref.	New T0	0		Ref.
1	1.59 (0.91-2.77)	New T1	1		1.59 (0.91-2.76)
2	0.75 (0.56-1.01)	New T2	2		0.75 (0.56-1.00)
3	0.40 (0.31-0.53)	New T3	3	CRM (-)	0.43 (0.33-0.56)
4A	0.17 (0.10-0.30)	New T4A	4A	CRM (-)	0.20 (0.11-0.38)
4B	0.12 (0.08-0.19)	New T4B	3	CRM (+)	0.15 (0.10-0.22)
			4B	CRM (-)	
		New T4C	4A	CRM (+)	0.10 (0.06-0.18)
			4B	CRM (+)	
*Harrell's C-Index (95%CI)*	*0.752 (0.735-0.770)*				*0.756 (0.738-0.773)*
*P-value*					*0.034*

Adjusted variable including age, sex, grade, pN, surgery type, LNR group, adjuvant treatment, and CCI score. Abbreviations: ypT, pathological tumor; CRM, circumferential resection margin.

**Table 5:** The performance of new category pT in rectal cancer patients treated with neoadjuvant chemoradiotherapy and surgery.

	Total patient	*Model 2: Harrell's C-Index*
Variable		Bootstrap Coef. (95% CIs)
CRM-inclusive ypT staging system	4308	*0.756 (0.720-0.791)*
*External validation*		
ypN		
-	2908	*0.722 (0.698-0.747)*
+	1400	*0.750 (0.723-0.777)*
LN removed number		
< 12	1518	*0.755 (0.721-0.789)*
≥ 12	2790	*0.761 (0.726-0.795)*

## Data Availability

Clinicopathological datasets are available from the corresponding author upon reasonable request.

## Abbreviations

LARC: locally advanced rectal cancer
NACRT: neoadjuvant chemoradiotherapy
TME: total mesorectal excision
TCR: Taiwan cancer registry
NHIRD: National Health Insurance Research Database
AJCC: American Joint Committee on Cancer
ICD-O-3: International Classification of Diseases for Oncology, 3rd Edition
CCI: Charlson comorbidity index
OR: Odds ratio
CI: confidence interval
