# Supplementary material for: A CRM-Integrated ypT Staging System Improves Prognostic Stratification Following Neoadjuvant Therapy in Rectal Cancer
Source: J Cancer. 2026 Mar 17;17(3):679–87. doi: 10.7150/jca.129779 (PMC13003605; doi:10.7150/jca.129779)

Supplement Table 1. Survival rates of patients with rectal cancer according to different ypT and CRM status.

	Survival			Survival	
***p*T**	**Total**	**N (%)**	***p*T with CRM**	**Total**	**N (%)**
*p*T0	756	666 (88.10)	*p*T0		
			CRM(-)	755	665 (88.08)
			CRM(+)	1	1 (100.00)
*p*T1	204	187 (91.67)	*p*T1		
			CRM(-)	235	215 (91.49)
			CRM(+)	1	1 (100.00)
*p*T2	1,037	876 (84.47)	*p*T2		
			CRM(-)	1,035	875 (84.54)
			CRM(+)	2	1 (50.00)
*p*T3	2,118	1,521 (71.81)	*p*T3		
			CRM(-)	1,986	1,460 (73.51)
			CRM(+)	132	61 (46.21)
*p*T4	193	94 (48.70)	*p*T4		
			CRM(-)	121	64 (52.89)
			CRM(+)	72	30 (41.67)

Supplement Figure 1. The ROC between ypT and new category pT in rectal cancer patients treated with neoadjuvant chemoradiotherapy and surgery.

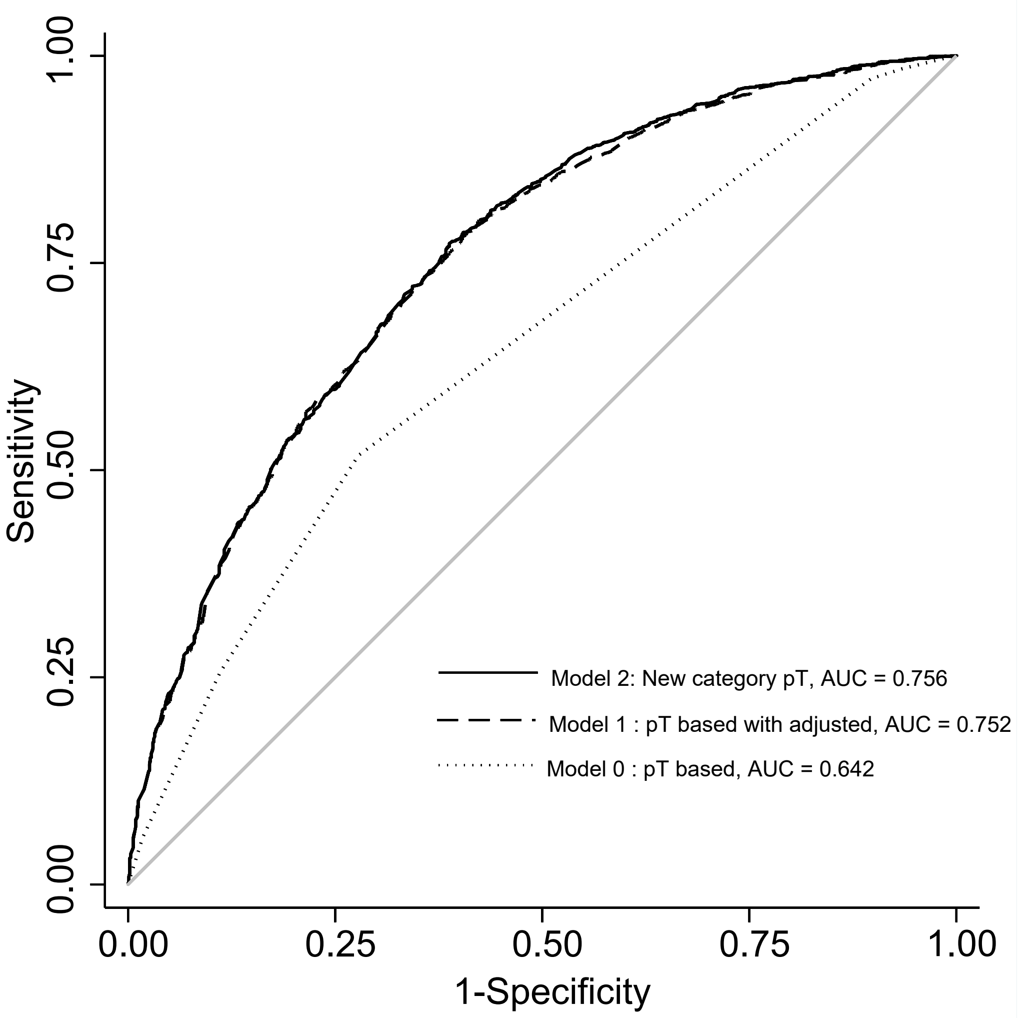

Supplement: Supplementary file 1 — Supplementary figure and table. [file jcav17p0679s1.pdf]
